# Infectious Disease: Pathogenesis, Prevention and Case Studies

**DOI:** 10.3201/eid1601.091408

**Published:** 2010-01

**Affiliations:** Centers for Disease Control and Prevention, Atlanta, Georgia, USA

**Keywords:** infectious disease, case studies, textbook, viruses, bacteria, parasites, book review

The organizing vision of this textbook is neither a taxonomic outline of the microbiologic world nor an epidemiologic understanding of our evolving insights into epidemics. Rather it is translational, ecologic, holistic, and distinctly clinical. It is a fun and readable book that engages the imagination and retains the interest of the clinically oriented reader while conveying an understanding of the direct implications of molecular characteristics of infectious agents to the practice of medicine.

The chapters in Part 4, Infections of Global Impact, and Part 5, Emerging and Resurgent Infections, are especially likely to fire the imaginations of students in introductory clinical microbiology or infectious disease classes. The chapters in Part 1, General Principles of Infectious Diseases, will equally effectively assist infectious disease professionals in mid to late career who seek an easy and enjoyable way to refresh and update their understanding of such topics as microbial structure, mechanisms of action of antimicrobial agents, and laboratory approaches to investigation and diagnosis.

That said, this is not a book for all audiences. The success with which it integrates the microbiologic world with the world of medical practice sacrifices approaches that might engage persons for whom the end point of microbiologic interest is not human disease. It is neither balanced nor comprehensive enough to function as a definitive reference. Also, chapters vary in quality. In the chapter on host defenses, for example, attempts to simplify occasionally lead to unfortunately loose statements such as “Within man, there are certain well known racial differences in disease susceptibility…,” among which it identifies “Dark skinned individuals have an increased susceptibility to coccidioidomycosis.” These statements confuse me.

I am aware that early 20th century surveys identified lower prevalence of hookworm disease among residents of the rural American South who are of African descent than among their neighbors of European descent despite similar living habits, environmental conditions, and levels of impoverishment. Accurately or not, these differences were attributed to racial variability in the effectiveness of skin as a barrier against larval hookworm invasion. But what can be the relevance of dark skin to an infection that invades primarily by inhalation of spores distributed in the soil of the American Southwest, Mexico, Central, and South America? In fact, susceptibility to primary coccidioidal infection is not affected by racial background. The frequency of dissemination is higher among Filipinos, Hispanics, and blacks. This frequency may reflect genetic host factors, or it may identify ethnicity or socioeconomic status as a marker for risk of environmental exposure to larger inocula. The jury is out.

Similar occasional failures to speak with precision distracted me and thus detracted from what is overall an excellent book. In contrast, the chapters on influenza, infections in the returning traveler (a tutorial in how to think like a travel medicine specialist), and emerging and resurgent infections were excellent.

